# Determinants of Carboxyhemoglobin Levels and Relationship with Sepsis in a Retrospective Cohort of Preterm Neonates

**DOI:** 10.1371/journal.pone.0161784

**Published:** 2016-08-23

**Authors:** Andrew J. McArdle, James Webbe, Kathleen Sim, Graham Parrish, Clive Hoggart, Yifei Wang, J. Simon Kroll, Sunit Godambe, Aubrey J. Cunnington

**Affiliations:** 1 Paediatric Infectious Diseases, Imperial College Healthcare NHS Trust, London, United Kingdom; 2 Neonatal Medicine, Imperial College Healthcare NHS Trust, London, United Kingdom; 3 Section of Paediatrics, Department of Medicine, Imperial College, London, United Kingdom; 4 Imperial College School of Medicine, South Kensington Campus, Imperial College, London, United Kingdom; University of Missouri Health Care, UNITED STATES

## Abstract

Carboxyhemoglobin levels in blood reflect endogenous carbon monoxide production and are often measured during routine blood gas analysis. Endogenous carbon monoxide production has been reported to be increased during sepsis, but carboxyhemoglobin levels have not been thoroughly evaluated as a biomarker of sepsis. We sought to determine whether carboxyhemoglobin levels were elevated during sepsis in a high risk population of premature neonates. We conducted a retrospective cohort study of 30 infants in two neonatal intensive care units using electronic medical and laboratory records. The majority of infants were extremely premature and extremely low birth weight, and 25 had at least one episode of sepsis. We collected all carboxyhemoglobin measurements during their in-patient stay and examined the relationship between carboxyhemoglobin and a variety of clinical and laboratory parameters, in addition to the presence or absence of sepsis, using linear mixed-effect models. We found that postnatal age had the most significant effect on carboxyhemoglobin levels, and other significant associations were identified with gestational age, hemoglobin concentration, oxyhemoglobin saturation, and blood pH. Accounting for these covariates, there was no significant relationship between the onset of sepsis and carboxyhemoglobin levels. Our results show that carboxyhemoglobin is unlikely to be a clinically useful biomarker of sepsis in premature infants, and raise a note of caution about factors which may confound the use of carbon monoxide as a clinical biomarker for other disease processes such as hemolysis.

## Introduction

Blood gas analysis is one of the main methods used to monitor the effectiveness of ventilation and metabolic status of preterm infants [1]. Many clinical blood gas analysers in current use have a co-oximetry module, used primarily by clinicians to give an estimate of hemoglobin concentration, but also reporting carboxyhemoglobin (COHb) and methemoglobin (MetHb) levels. Consequently many co-oximetry measurements are performed on sick neonates although the COHb levels are rarely noted by clinicians. The recognition of endogenously produced carbon monoxide (CO) as a molecule with unique physiological properties [2, 3], and as a clinically useful indicator of hemolysis and other pathological states, has renewed interest in methods for its detection [4–7].

Endogenous CO production mainly arises from heme catabolism by heme oxygenase (HO) enzymes, along with two other degradation products: iron and biliverdin [8]. The inducible isoform of HO is HO-1 [9], and aside from its role in heme catabolism it has also been shown to have an indispensable cytoprotective role in the cellular response to a wide variety of harmful stimuli [10, 11] including an essential function for survival in animal infection models such as malaria and bacterial sepsis [12–14]. HO-1 is encoded by the *HMOX1* gene in humans located on chromosome 22 q12 [15] and is largely regulated at the transcriptional level [16–18]. An increase in heme degradation by HO-1 results in a detectable increase in CO production, blood levels of COHb, and CO excretion in breath [19–21]. This increase in CO production has been demonstrated in a variety of hemolytic anemias, including hemolytic disease of the newborn and ABO incompatibility [4, 7, 19, 21]. In addition, increased CO production has been observed following non-heme induction of HO-1, for example during the systemic inflammatory response syndrome due to trauma or infection [22–26].

Neonatal sepsis causes a huge burden of disease globally [27]. Even in well-resourced settings, early-onset sepsis remains a significant cause of neonatal morbidity and mortality, whilst late-onset sepsis, particularly nosocomial sepsis accompanying neonatal intensive care for prematurely born infants, is a major problem [28]. Very low birth weight (VLBW, <1500g) infants are at high risk of developing sepsis in the neonatal period and using conservative estimates, at least 15% will develop culture-proven sepsis [28]. Many more infants will be suspected to have bacterial sepsis based on clinical features and laboratory investigations, but blood cultures will not yield an organism. Furthermore, the diagnosis of sepsis is often complicated because traditional measures of the host-response to infection, such as C-reactive protein and white blood cell count, may lack sensitivity in neonates [28, 29]. For this reason, new methods of identifying the onset of sepsis are highly desirable [30], and might allow more rational use of antibiotics—avoiding selection of resistant organisms and changes in the early gut microbiota [31]. We wondered if COHb might be a useful indicator of the onset of sepsis in neonates.

The aim of the present study was to identify patient characteristics and physiological parameters associated with COHb concentrations in a retrospective cohort of neonates admitted to two neonatal units in the UK, and then test the hypothesis that COHb concentrations were increased at the onset of episodes of sepsis. We identified multiple determinants of COHb, with postnatal age having the strongest effect, although the onset of sepsis did not appear to significantly influence COHb concentrations. Nevertheless, these results will be useful when considering interpretation of COHb levels for other purposes such as identification of pathological hemolysis.

## Materials and Methods

### Study design and subject identification

The study was approved by the National Research Ethics Committee South Central—Hampshire A (Reference 15_SC_0263) and the Joint Research Compliance Office of Imperial College London and Imperial College Healthcare NHS Trust (ICHT). We performed a retrospective analysis using prospectively collected electronic routine clinical and laboratory data from the neonatal units at St Mary’s and Queen Charlotte’s Hospitals, which are both part of ICHT. To minimise any inclusion bias, individual consent was not obtained for access to the health records, which was acceptable because records were only accessed by members of the patient care team, and only pseudoanonymised data was used for analysis. The Vermont Oxford Network 2014 Manual of Operations Part 2, Release 18.0 (https://public.vtoxford.org/membership/member-tools/manuals-forms/) was used to define all clinical events, since this was the basis for recording outcomes in the neonatal electronic records. Any infant admitted on the neonatal units at St Mary’s Hospital or Queen Charlotte’s Hospital between June 2014 and June 2015 with at least two COHb measurements was potentially eligible for inclusion. Exclusion criteria were: proven or suspected hemolytic disorder (increases COHb); necrotizing entercolitis (from 24 hours prior to clinical suspicion; increased HO-1 expression [32] is likely to increase COHb and confound identification of sepsis); nitric oxide therapy or therapeutic cooling (for the duration of this therapy because effect on COHb is unknown); major congenital malformations (may influence clinical suspicion of sepsis). The group of infants with at least one episode of sepsis was composed of all infants who met the Vermont Oxford Network definition of early or late sepsis and had at least one COHb measurement at “onset” of sepsis (defined as the interval from six hours before the sample yielding a positive culture was obtained or six hours before starting treatment, until 24hrs after start of appropriate antimicrobial treatment). A second group of infants without sepsis was identified from all infants who never met Vermont Oxford Network definition of early or late sepsis, and did not receive any antibiotic treatment for suspected sepsis after the first 48 hours of life (the period in which almost all preterm neonates received empirical antibiotic cover according to unit protocols). Infants without sepsis were selected from all eligible patients by matching gestational age to a random selection of infants with sepsis.

#### Data collection and analysis

Blood gas analysis and co-oximetry was performed using GEM4000 analysers (Instrumentation Laboratory) and results were automatically transferred to the hospital electronic laboratory record system. Electronic laboratory results together with the time and date of sample collection were extracted using CERNER Powerchart (CERNER corporation). Demographic and clinical data was extracted from the BadgerNet neonatal electronic patient records (Clevermed). We collected information on the following parameters: organisms grown from sterile site cultures; blood gas pH, COHb (%), oxyhemoglobin (%), methemoglobin (%), hemoglobin concentration; neonatal unit (St Mary’s or Queen Charlotte’s Hospital); gestation; birth weight; gender; clinical diagnoses; blood transfusions; survival to discharge. Potential episodes of sepsis were identified from the diagnoses recorded in the electronic patient records and an additional database of all bacteremias arising from the neonatal units during the study period which was provided by the microbiology laboratory. Local hourly atmospheric CO measurements were obtained from the Department for the Environment and Rural Affairs (http://uk-air.defra.gov.uk/). We defined the following terms prior to analysis: onset of sepsis, as above; duration of sepsis, from onset of sepsis until antimicrobials were stopped or death or discharge; suspected sepsis, any episode where there was antimicrobial treatment but the Vermont Oxford Network definitions of early or late sepsis were not met; no evidence of sepsis, absence of sepsis and suspected sepsis; recent blood transfusion, blood transfusion on the same day or day prior to the sample collection. We used all data for analysis without exclusion of outliers (although the accuracy of any apparent outlying results was verified against the original patient or laboratory records). Missing data was excluded with the exception of environmental CO levels where we interpolated missing values for up to four one-hour time periods by assuming a linear trend between the closest time points for which measurements were available.

Data were analysed in the R statistical programme using the packages lme4 and lmertest [33]. In a pilot study we had found that trends in repeated measurements of COHb over time were influenced by multiple factors including postnatal age, weight, oxyhemoglobin %, pH, and subject identity. For this reason, our primary analysis was performed using a generalised linear mixed-effects model, which allows for multiple covariates to be included and the effect of sepsis to be determined in relation to the effect of these covariates. Subject identity was included as a random factor, and the model was refined by step-wise removal of the least significant variables from a list of potentially relevant covariates until all variables in the model remained significant (*P* <0.05). In order to determine the most important covariates this process was first applied to data collected from all subjects at times when there was no suspected or proven sepsis. The process was then repeated incorporating data collected at the onset of sepsis in addition to the data collected when there was no evidence of sepsis. In simple terms, this approach allows multiple measurements on each individual to be used, such that changes in COHb can be assessed relative to the other measurements for that individual, but it also allows the data on multiple individuals (both with and without sepsis, and at non-standardised time points) to be combined in order to increase statistical power to detect effects of covariates. Based on the numbers of neonates with sepsis seen each month in the neonatal units we expected to be able to identify approximately 20 neonates fulfilling the sepsis definition from a one-year period. Strictly speaking, this method of analysis does not require a control group because longitudinal measurements of COHb allow each individual to act as their own control, by comparing measurements during septic episodes with those in the absence of sepsis. Therefore we arbitrarily included data only 5 additional subjects who had no episodes of sepsis.

Subject data are included as Supporting Information, S1 Table.

## Results

### Characteristics of study subjects

Fifty infants with a diagnosis of sepsis were screened for eligibility, of whom twenty-five fulfilled the inclusion criteria for the sepsis group and did not have any exclusion criteria. Sixty-three infants were screened for inclusion in the no sepsis group, of whom only five met the strict inclusion criteria and had no exclusion criteria. Characteristics of the subjects are shown in Table 1. Amongst the twenty-five neonates with sepsis twenty-two had one episode of sepsis, and three had two episodes of sepsis. In all episodes of sepsis the pathogens were primarily isolated from the blood, and no episodes were defined on the basis of a positive culture from another sterile site without bacteremia. The distribution of causative organisms and the timing of onset of infection is shown in Table 2.

**Table 1:** Characteristics of study subjects.

	Sepsis (n = 25)	No sepsis (n = 5)
Gestational age, completed weeks + days. Median (range)	25+0 (23+2 to 34+5)	25+3 (25+2 to 27+0)
Birth weight, g.Median (range)	735 (460–1885)	740 (692–820)
Male, n (%)	14 (56%)	0 (0%)
Antenatal glucocorticoid, n (%)	23 (92%)	5 (100%)
Vaginal delivery, n (%)	17 (68%)	4 (80%)
Length of stay on neonatal unit, days. Median (range)	48.5 (1–392)	20 (3–78)
Number of carboxyhemoglobin measurements.Median (range)	34 (3–145)	17 (5–29)
At least one blood transfusion, n (%)	21 (84%)	4 (80%)
Respiratory distress syndrome, n (%)	24 (96%)	5 (100%)
Hypoxic ischemic encephalopathy, n (%)	0 (0%)	1 (20%)
Survived to discharge, n (%)	18 (72%)	2 (40%)

**Table 2:** Characteristics of sepsis episodes.

	Early Onset (n = 4)	Late Onset (n = 24)
Group B *Streptococcus*	2	2
*Escherichia coli*	1	1
Coagulase-negative Staphylococci	0	17
*Staphylococcus aureus*	0	2
Other organisms	1 (*Streptococcus gallolyticus*)	2 (*Acinetobacter* species, *Pseudomonas aeruginosa*)
Survived, n (%)	3 (75%)	20 (83%)
Duration of sepsis in survivors, days. Median (range)	14 (5–14)	5 (5–14)

### Determinants of carboxyhemoglobin levels

**Fig 1:**
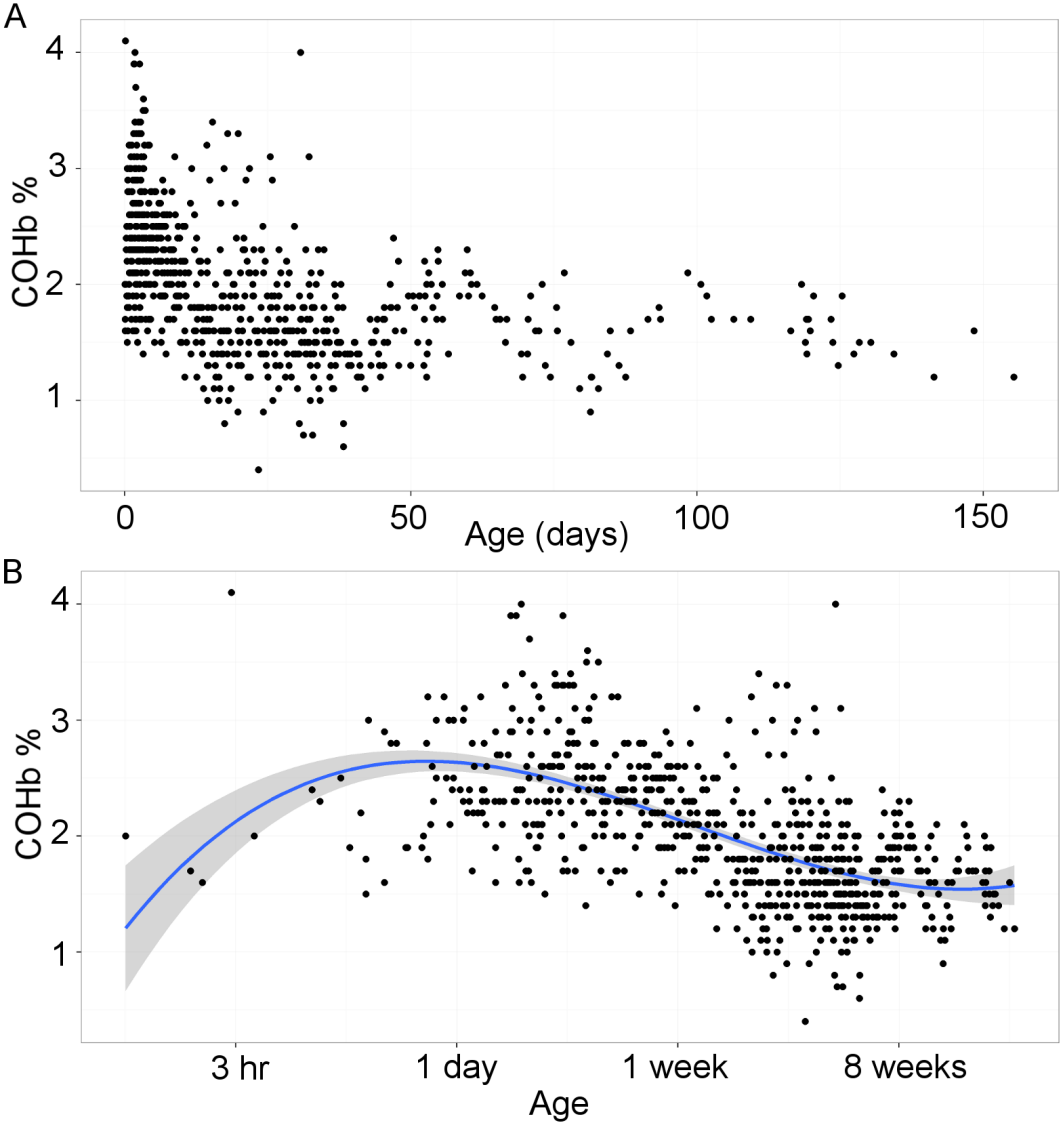
Variation in carboxyhemoglobin levels with postnatal age. (A) Carboxyhemoglobin levels plotted against postnatal age on a linear scale. (B) Carboxyhemoglobin levels plotted against postnatal age on a logarithmic scale. Solid line indicates the line of best fit using a third order polynomial (cubic) function and shaded region indicates 95% confidence interval.

In a pilot study we observed that COHb concentrations in neonates were strongly related to postnatal age, so we began by examining this association in order to identify the best way to handle age in our model. Restricting analysis to data collected when there was no evidence of sepsis, we identified that COHb concentration declined with time from birth (Fig 1A). However closer inspection with postnatal age plotted on a logarithmic scale revealed that the relationship was more complex (Fig 1B). There appeared to be an initial increase in COHb reaching a peak about one day after birth, followed by a decrease until a steady state was reached after about 8 weeks. This trend appeared to be well described by a third order polynomial expression, and indeed this produced a much better explanation of the data than a linear or quadratic solution, as demonstrated by the lower Akaike Information Criterion (AIC) and Bayesian Information Criterion (BIC) (Table 3). Using this relationship to account for the effect of age we constructed a linear mixed-effects model to identify covariates which might significantly influence COHb concentration when there was no evidence of sepsis. This revealed that COHb was positively associated with oxyhemoglobin saturation and hemoglobin concentration, and negatively associated with gestational age and blood pH (Table 4). Of note, COHb was not significantly associated with atmospheric CO, recent blood transfusion or neonatal outcome (survival or death).

**Table 3:** Comparison of models to explain the association of postnatal age with carboxyhemoglobin levels.

Model and variables†	Akaike Information Criterion	Bayesian Information Criterion	Log likelihood	Chi- squared	*P* (for indicated comparisons)
0. Intercept only (null)	1211	1225	-603	Reference	
1. Age	1013	1031	-502	201	< 2.2 x10^-16^ vs. model 0
2. ln (Age)	891	909	-441	121	< 2.2 x10^-16^ vs. model 0
3. ln (Age) + (ln (Age))^2^	852	875	-421	41.1	1.48x10^-10^ vs. model 2
4. ln (Age) + (ln (Age))^2^ + (ln (Age))^3^	814	841	-401	40.2	2.35x10^-10^ vs. model 3

^†^All models include subject identity as a random factor.

**Table 4:** Linear mixed-effects model to describe the effect of variables on COHb levels in the absence of sepsis.

Variables included in model†	Estimate (standard error)	t value	*P*
ln Age	-4.97 x10^-2^ (2.91 x10^-2^)	-1.71	0.087
(ln Age)^2^	-0.11 (1.47 x10^-2^)	-7.13	2.68 x10^-12^
(ln Age)^3^	1.48 x10^-2^ (2.75 x10^-3^)	5.39	9.77 x10^-8^
Oxyhemoglobin (%)	6.54 x10^-3^ (1.67 x10^-3^)	3.92	9.83 x10^-5^
Gestational age (weeks)	-8.45 x10^-2^ (1.66 x10^-2^)	-5.09	3.55 x10^-5^
pH	-0.53 (0.23)	2.32	0.020
Hemoglobin g/L	1.92 x10^-3^ (7.39 x10^-4^)	2.60	9.49 x10^-3^
Variables eliminated from model			
Unit (St Mary’s / QCCH)			NS
Atmospheric CO (ppm)			NS
Gender			NS
Methemoglobin (%)			NS
Birthweight (g)			NS
Recent blood transfusion			NS
Outcome (survived/died)			NS

^†^Based on 674 observations; all models include subject identity as a random factor. NS, not significant (*P*>0.05) and therefore removed sequentially from model.

### Relationship between onset of sepsis and carboxyhemoglobin levels

**Fig 2:**
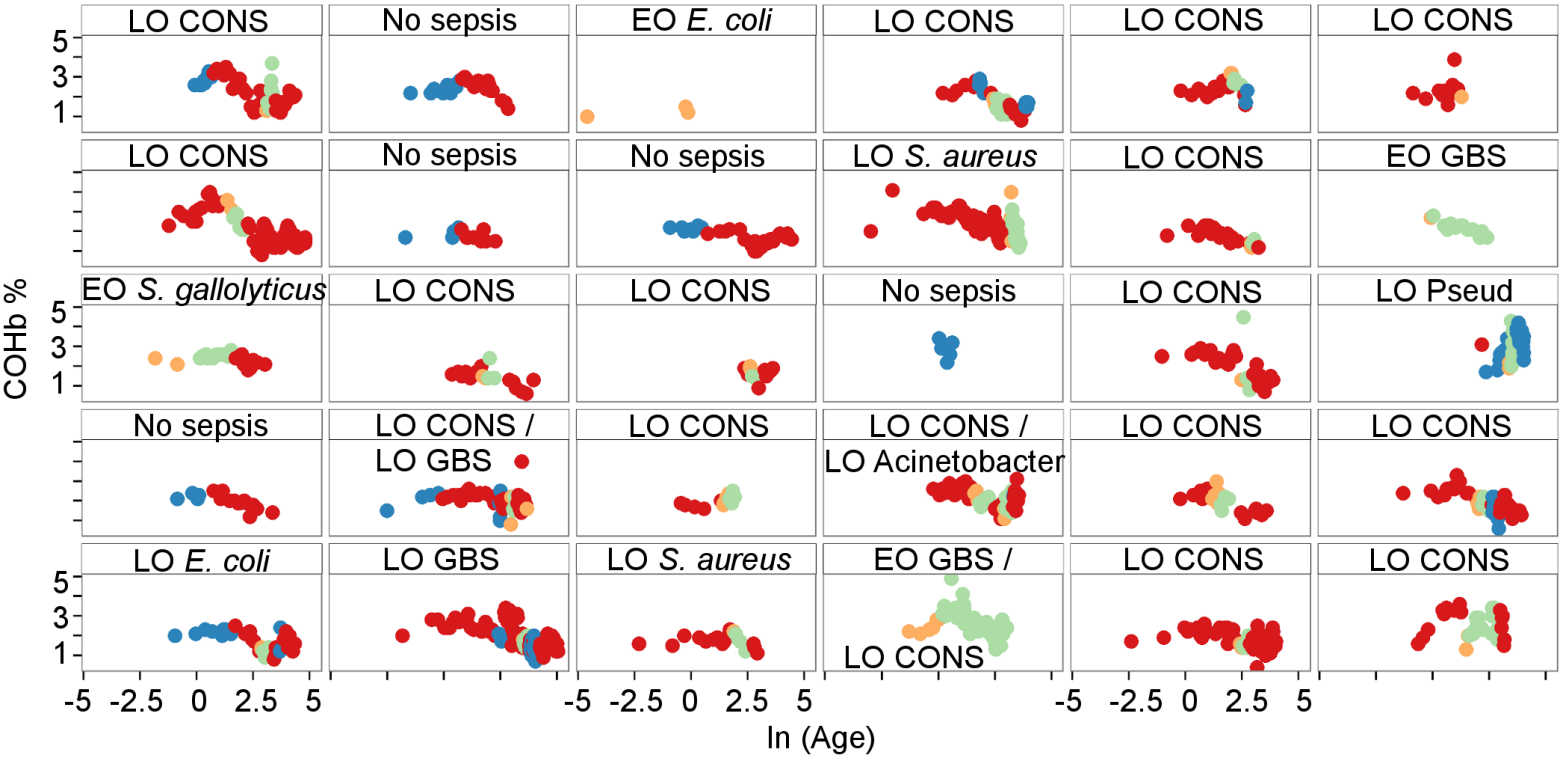
Carboxyhemoglobin levels in individual subjects in relation to postnatal age and episodes of sepsis. Trellis plot of COHb levels over time (ln (Age)) for each individual subject and relationship to presence or absence of sepsis (red, no sepsis; blue, suspected sepsis; orange, onset of proven sepsis; green, established sepsis). Timing and cause of sepsis are indicated for each individual (EO, early onset; LO, late onset; GBS, Group B Streptococcus; CONS, coagulase-negative Staphylococcus; Pseud, *Pseudomonas aeruginosa*; Acinetobacter, *Acinetobacter* sp.)

We repeated the process described above to evaluate if onset of sepsis was significantly associated with COHb levels in the linear mixed-effects model. Onset of sepsis was not associated with any significant difference in COHb, and the best model (Table 5) remained very similar to the model which only included measurements taken in the absence of sepsis. We repeated this process to see whether any effect could be detected if the cause of sepsis was considered. Since coagulase negative Staphylococci (CONS) often cause a more insidious illness (usually associated with infection of a central venous catheter) we explored whether onset of sepsis due to CONS or non-CONS organisms may show differential association with COHb. This was not the case. We also explored whether COHb may be differentially associated with early or late onset sepsis, but again we found no significant associations. Overall this suggests that COHb is not significantly different at the onset of sepsis when other covariates are taken into account. We inspected the data from each individual subject to examine whether there was any obvious relationship between COHb levels and episodes of sepsis (Fig 2). It was clear from this data that in some cases elevated levels of COHb did occur during episodes of sepsis, but there was no consistent temporal pattern in relation to the start of the episode of sepsis. Overall, neonates with and without sepsis seemed to have similar patterns of COHb with respect to age.

**Table 5:** Linear mixed-effects model to describe the effect of variables on COHb levels including data at the onset of sepsis.

Variables included in model†	Estimate (standard error)	t value	*P*
ln Age	-6.55 x10^-2^ (2.71 x10^-2^)	-2.42	0.016
(ln Age)^2^	-6.53 x10^-2^ (9.34 x10^-3^)	-6.99	6.41 x10^-12^
(ln Age)^3^	7.23 x10^-3^ (2.01 x10^-3^)	3.61	3.33 x10^-4^
Oxyhemoglobin (%)	5.57 x10^-3^ (1.73 x10^-3^)	3.22	1.33 x10^-3^
Gestational age (weeks)	-7.38 x10^-2^ (1.68 x10^-2^)	-4.40	2.18 x10^-4^
pH	-0.52 (0.22)	-2.34	0.019
Hemoglobin g/L	2.13 x10^-3^ (7.42 x10^-4^)	2.87	4.17 x10^-3^
Methemoglobin (%)	0.17 (5.35 x10^-2^)	2.18	0.030
Variables eliminated from model			
Onset of sepsis			NS
Unit (St Mary’s / QCCH)			NS
Atmospheric CO (ppm)			NS
Gender			NS
Birthweight (g)			NS
Recent blood transfusion			NS
Outcome (survived/died)			NS

^†^Based on 733 observations; all models include subject identity as a random factor. NS, not significant (*P*>0.05) and therefore removed sequentially from model.

## Discussion and Conclusion

The potential to identify hemolysis from detection of increased endogenous CO production has been noted since at least 1949 [19, 34–36]. The realization that CO production also increases in other circumstances when there is HO-1 induction, such as infection and systemic inflammation [23–26], has also raised the possibility that CO may be a useful biomarker of these conditions. Early diagnosis of infection in premature neonates can be challenging and numerous biomarkers have been examined without any yet achieving the perfect combination of being cheap and simple to measure whilst highly sensitive and specific [29, 37]. Since COHb is so frequently measured during routine blood gas analysis in neonatal units, noting a sudden change or sustained trend in COHb levels might aid the recognition of a new disease process. In order to assess whether COHb could be clinically useful as a biomarker of sepsis or hemolysis we need to understand which other factors influence COHb levels. Here we examined a variety of factors which might influence COHb levels in a longitudinal dataset from preterm neonates, and we tested whether COHb levels were significantly altered by the onset of sepsis. Although we found that the onset of sepsis did not influence COHb concentrations, we did characterise several other variables with an important influence on COHb levels. Most notable of these was postnatal age, which will need to be accounted for in any studies investigating the association between COHb and clinical diagnoses or outcomes.

Our study was conducted in a population predominantly composed of extremely preterm and extremely low birth weight neonates, the most vulnerable population for risk of infection [28]. We found that COHb levels increased over approximately the first 24 hours after birth, before declining over the next 8 weeks to a steady value. The initial increase in COHb could reflect the change from feto-placental to postnatal circulation, because the CO binding affinity of fetal hemoglobin is lower than that of hemoglobin A [38]. Thus *in utero* CO might preferentially cross the placenta to the maternal blood, resulting in levels of COHb in the neonate immediately after birth which are lower than expected for the amount of endogenous CO production. Other possible explanations include an increase in HO-1 expression in response to birth [39], and/or an increase in the rate of heme catabolism from fetal red blood cells after birth. Following the initial rise, COHb levels declined over a time course reminiscent of the natural fall in hemoglobin concentration after birth [40]. The shortened lifespan of fetal red blood cells, particularly those from preterm infants [40], might perhaps explain this through an increased rate of heme catabolism until these cells are eventually all removed from the circulation. Finally a steady state level of COHb was reached at around 8 weeks, which is close to the expected nadir for hemoglobin concentration [40]. COHb was positively associated with oxyhemoglobin saturation, as we previously observed in a cross-sectional study of children in Kenya [22], and this likely represents the fact that at low concentrations of both CO and oxygen there is enhancement of binding to hemoglobin [41, 42]. We also found that COHb levels were inversely correlated with gestational age (as previously noted by others [43]), and positively associated with hemoglobin concentration, two observations which may both be related to the generation of CO from more rapid destruction of fetal red cells after birth. Prematurity is associated with a lower hemoglobin concentration and red cell mass at the time of birth [44], and the fetal red cell mass dictates the amount of heme which will be catabolised during the shorter lifespan of fetal erythrocytes [40]. The negative association of COHb with pH was unexpected, since low pH has been reported to enhance CO dissociation from hemoglobin [45], however acidosis can also induce HO-1 expression [46]. The positive association with methemoglobin (at least in the analysis including onset of sepsis) is not surprising since methemoglobin formation precedes the liberation of heme from hemoglobin, and hence increased methemoglobin is likely to indicate increased heme availability and therefore CO production [47, 48]. We did not find any association of COHb with onset of sepsis, suggesting that COHb would not be a useful biomarker of sepsis in this population.

Our study has a number of limitations, primarily due to its retrospective nature and the constraints this imposes on data availability and data quality. We did not have any control over the timing of samples or the reason for blood sampling, which means that samples were most likely to be collected when infants were most unwell. We did not have reliable data to indicate whether samples were arterial, venous or capillary in origin, which may be important because differences in arterial and venous COHb have been reported to occur and to be greater in the presence of lung disease [49, 50]. However we believe this would not have a major confounding effect because common practice in our neonatal unit is to collect capillary samples, with a smaller proportion of venous origin, and very few of arterial origin. We were unable to extract accurate information about artificial ventilation parameters and supplemental oxygen concentrations from the electronic patient records and so were unable to adjust for these in our analysis. Another limitation is the use of COHb levels measured on a blood co-oximeter, and the assumption that this reflects endogenous CO production. More accurate methods exist for determination of CO content in blood [51], and for measurement of endogenous CO production [52], but none of these are practical to apply to a longitudinal study with repeated samples collected from extremely small and often very ill neonates. Our approach was deliberately pragmatic, since we sought to identify whether the COHb levels which are routinely measured as part of blood gas analysis were of any utility for the identification of sepsis.

Our study is not the first to examine COHb levels in neonates, but we believe that it provides the most complete characterization of COHb levels over time after birth, and it is the first to examine whether the onset of sepsis is independently associated with COHb levels. In contrast to another study we found no association of COHb levels with survival [43], but this may simply reflect the smaller number of infants in our study. The lack of an increase in COHb at the onset of sepsis in our study may reflect the nature of the inflammatory response to sepsis in extremely premature infants which also results in reduced sensitivity of conventional markers of sepsis [28, 29]. We excluded infants with necrotising enterocolitis from our study in order to prevent it confounding relationships between COHb and sepsis, but the relationships between COHb and necrotizing enterocolitis may be interesting to study in their own right. It is known that HO-1 expression is increased in the intestines of infants with necrotising enterocolitis, whilst carbon monoxide protects from necrotising enterocolitis-like pathology in neonatal rats [32]. Therefore future studies may be justified to examine whether COHb levels in extremely preterm infants have value in predicting risk or making an early diagnosis of necrotising enterocolitis.

Our findings have several implications. First the raw data themselves (Fig 2) highlight the considerable variability of COHb levels between and within individuals. We have characterised some variables which account for some of this variation, most notably postnatal age. These variations suggest that tests for hemolysis or other pathological processes based on measurement of exhaled CO or COHb may not perform as well in a real world setting as they might in carefully controlled trials. Accounting for the variations due to postnatal age, pH, hemoglobin, oxyhemoglobin, and gestational age will introduce a level of complexity which is difficult to adapt to clinical practice. However knowledge of these potential confounding variables may be useful in future studies seeking to identify biological relationships between COHb and pathological processes, for example necrotising entercolitis as mentioned above. Whilst we found no association between onset of sepsis and COHb, inspection of the data in Fig 2 does suggest that some of the highest levels of COHb tended to occur during episodes of sepsis. It would therefore be prudent for clinicians to be aware of possible reasons for elevated COHb in a neonate, and to consider carefully whether there may be an underlying reason.

## Supporting Information

S1 Table Subject data. (XLSX)

## References

[1] Tan RN, Mulder EE, Lopriore E, Te Pas AB. Monitoring Oxygenation and Gas Exchange in Neonatal Intensive Care Units: Current Practice in the Netherlands. Front Pediatr. 2015;3:94 10.3389/fped.2015.00094 26579504 PMC4630576

[2] Wu L, Wang R. Carbon monoxide: endogenous production, physiological functions, and pharmacological applications. Pharmacol Rev. 2005;57(4):585–630. Epub 2005/12/31. 57/4/585 [pii] 10.1124/pr.57.4.3 . 16382109

[3] Motterlini R, Otterbein LE. The therapeutic potential of carbon monoxide. Nat Rev Drug Discov. 2010;9(9):728–43. 10.1038/nrd3228 . 20811383

[4] Tidmarsh GF, Wong RJ, Stevenson DK. End-tidal carbon monoxide and hemolysis. J Perinatol. 2014;34(8):577–81. 10.1038/jp.2014.66 . 24743136

[5] Caboot JB, Jawad AF, McDonough JM, Bowdre CY, Arens R, Marcus CL, et al Non-invasive measurements of carboxyhemoglobin and methemoglobin in children with sickle cell disease. Pediatric Pulmonol. 2012;47(8):808–15. 10.1002/ppul.22504 22328189 PMC3368100

[6] Ryter SW, Choi AM. Carbon monoxide in exhaled breath testing and therapeutics. J Breath Res. 2013;7(1):017111 10.1088/1752-7155/7/1/017111 23446063 PMC3651886

[7] Lozar-Krivec J, Bratanic B, Paro-Panjan D. The role of carboxyhemoglobin measured with CO-oximetry in the detection of hemolysis in newborns with ABO alloimmunization. J Mat Fetal Neonatal Med. 2016;29(3):452–6. 10.3109/14767058.2015.1004050 . 25604086

[8] Tenhunen R, Marver HS, Schmid R. The enzymatic conversion of heme to bilirubin by microsomal heme oxygenase. Proc Natl Acad Sci U S A. 1968;61(2):748–55. Epub 1968/10/01. . 4386763 10.1073/pnas.61.2.748 PMC225223

[9] Maines MD, Trakshel GM, Kutty RK. Characterization of two constitutive forms of rat liver microsomal heme oxygenase. Only one molecular species of the enzyme is inducible. J Biol Chem. 1986;261(1):411–9. Epub 1986/01/05. . 3079757

[10] Ryter SW, Alam J, Choi AM. Heme oxygenase-1/carbon monoxide: from basic science to therapeutic applications. Physiol Rev. 2006;86(2):583–650. Epub 2006/04/08. 86/2/583 [pii] 10.1152/physrev.00011.2005 . 16601269

[11] Gozzelino R, Jeney V, Soares MP. Mechanisms of cell protection by heme oxygenase-1. Annu Rev Pharmacol Toxicol. 2010;50:323–54. Epub 2010/01/09. 10.1146/annurev.pharmtox.010909.105600 . 20055707

[12] Pamplona A, Ferreira A, Balla J, Jeney V, Balla G, Epiphanio S, et al Heme oxygenase-1 and carbon monoxide suppress the pathogenesis of experimental cerebral malaria. Nat Med. 2007;13(6):703–10. Epub 2007/05/15. nm1586 [pii] 10.1038/nm1586 . 17496899

[13] Seixas E, Gozzelino R, Chora A, Ferreira A, Silva G, Larsen R, et al Heme oxygenase-1 affords protection against noncerebral forms of severe malaria. Proc Natl Acad Sci U S A. 2009;106(37):15837–42. Epub 2009/08/27. 0903419106 [pii] 10.1073/pnas.0903419106 19706490 PMC2728109

[14] Larsen R, Gozzelino R, Jeney V, Tokaji L, Bozza FA, Japiassu AM, et al A central role for free heme in the pathogenesis of severe sepsis. Sci Transl Med. 2010;2(51):51ra71 Epub 2010/10/01. 2/51/51ra71 [pii] 10.1126/scitranslmed.3001118 . 20881280

[15] Kutty RK, Kutty G, Rodriguez IR, Chader GJ, Wiggert B. Chromosomal localization of the human heme oxygenase genes: heme oxygenase-1 (HMOX1) maps to chromosome 22q12 and heme oxygenase-2 (HMOX2) maps to chromosome 16p13.3. Genomics. 1994;20(3):513–6. Epub 1994/04/01. S0888-7543(84)71213-4 [pii] 10.1006/geno.1994.1213 . 8034330

[16] Alam J, Cook JL. How many transcription factors does it take to turn on the heme oxygenase-1 gene? Am J Respir Cell Mol Biol. 2007;36(2):166–74. Epub 2006/09/23. 2006-0340TR [pii] 10.1165/rcmb.2006-0340TR . 16990612

[17] Igarashi K, Sun J. The heme-Bach1 pathway in the regulation of oxidative stress response and erythroid differentiation. Antioxid Redox Signal. 2006;8(1–2):107–18. Epub 2006/02/21. 10.1089/ars.2006.8.107 . 16487043

[18] Sun J, Hoshino H, Takaku K, Nakajima O, Muto A, Suzuki H, et al Hemoprotein Bach1 regulates enhancer availability of heme oxygenase-1 gene. EMBO J. 2002;21(19):5216–24. Epub 2002/10/03. . 12356737 10.1093/emboj/cdf516 PMC129038

[19] Coburn RF, Williams WJ, Forster RE. Effect of Erythrocyte Destruction on Carbon Monoxide Production in Man. J Clin Invest. 1964;43:1098–103. 10.1172/JCI104994 14171787 PMC289591

[20] Engel RR, Rodkey FL, Krill CE Jr. Carboxyhemoglobin levels as an index of hemolysis. Pediatrics. 1971;47(4):723–30. . 5104458

[21] Lal A, Patterson L, Goldrich A, Marsh A. Point-of-care end-tidal carbon monoxide reflects severity of hemolysis in sickle cell anemia. Pediatr Blood Cancer. 2015;62(5):912–4. 10.1002/pbc.25447 . 25683629 PMC4376621

[22] Cunnington AJ, Kendrick SF, Wamola B, Lowe B, Newton CR. Carboxyhemoglobin levels in Kenyan children with Plasmodium falciparum malaria. Am J Trop Med Hyg. 2004;71(1):43–7. Epub 2004/07/09. 71/1/43 [pii]. . 15238687

[23] Moncure M, Brathwaite CE, Samaha E, Marburger R, Ross SE. Carboxyhemoglobin elevation in trauma victims. J Trauma. 1999;46(3):424–7. Epub 1999/03/24. . 10088844 10.1097/00005373-199903000-00012

[24] Shi Y, Pan F, Li H, Pan J, Qin S, Jiang D, et al Carbon monoxide concentrations in paediatric sepsis syndrome. Arch Dis Child. 2003;88(10):889–90. 14500308 10.1136/adc.88.10.889 PMC1719314

[25] Morimatsu H, Takahashi T, Matsusaki T, Hayashi M, Matsumi J, Shimizu H, et al An increase in exhaled CO concentration in systemic inflammation/sepsis. J Breath Res. 2010;4(4):047103 10.1088/1752-7155/4/4/047103 . 21383490

[26] Langeroudi AG, Hirsch CM, Estabragh AS, Meinardi S, Blake DR, Barbour AG. Elevated carbon monoxide to carbon dioxide ratio in the exhaled breath of mice treated with a single dose of lipopolysaccharide. Open Forum Infect Dis. 2014;1(2):ofu085 10.1093/ofid/ofu085 25734151 PMC4281777

[27] GBD 2103 Mortality and Causes of Death Collaborators. Global, regional, and national age-sex specific all-cause and cause-specific mortality for 240 causes of death, 1990–2013: a systematic analysis for the Global Burden of Disease Study 2013. Lancet. 2014 10.1016/S0140-6736(14)61682-2 . 25530442 PMC4340604

[28] Shane AL, Stoll BJ. Recent developments and current issues in the epidemiology, diagnosis, and management of bacterial and fungal neonatal sepsis. Am J Perinatol. 2013;30(2):131–41. 10.1055/s-0032-1333413 . 23297182

[29] Benitz WE. Adjunct laboratory tests in the diagnosis of early-onset neonatal sepsis. Clin Perinatol. 2010;37(2):421–38. 10.1016/j.clp.2009.12.001 . 20569816

[30] Ng PC, Ma TP, Lam HS. The use of laboratory biomarkers for surveillance, diagnosis and prediction of clinical outcomes in neonatal sepsis and necrotising enterocolitis. Arch Dis Child Fetal Neonatal Ed. 2015 10.1136/archdischild-2014-307656 . 25555389

[31] Mueller NT, Bakacs E, Combellick J, Grigoryan Z, Dominguez-Bello MG. The infant microbiome development: mom matters. Trends Mol Med. 2015;21(2):109–17. 10.1016/j.molmed.2014.12.002 25578246 PMC4464665

[32] Zuckerbraun BS, Otterbein LE, Boyle P, Jaffe R, Upperman J, Zamora R, et al Carbon monoxide protects against the development of experimental necrotizing enterocolitis. Am J Physiol Gastrointest Liver Physiol. 2005;289(3):G607–13. 10.1152/ajpgi.00055.2005 . 15890710

[33] Douglas B, Maechler M., Bolker B., Walker S. Fitting Linear Mixed-Effects Models Using lme4. Journal of Statistical Software. 2015;67(1):1–48. 10.18637/jss.v067.i01

[34] Coburn RF, Williams WJ, Kahn SB. Endogenous carbon monoxide production in patients with hemolytic anemia. J Clin Invest. 1966;45(4):460–8. 10.1172/JCI105360 5937023 PMC292720

[35] Engstedt L. Endogenous formation of carbon monoxide in hemolytic disease; with special regard to quantitative comparisons to other hemolytic indices. Acta Med Scand Suppl. 1957;332:1–63. . 13508119

[36] Sjostrand T. Endogenous formation of carbon monoxide in man. Nature. 1949;164(4170):580 . 18148861 10.1038/164580a0

[37] Simonsen KA, Anderson-Berry AL, Delair SF, Davies HD. Early-onset neonatal sepsis. Clin Microbiol Rev. 2014;27(1):21–47. 10.1128/CMR.00031-13 24396135 PMC3910904

[38] Engel RR, Rodkey FL, O'Neal JD, Collison HA. Relative affinity of human fetal hemoglobin for carbon monoxide and oxygen. Blood. 1969;33(1):37–45. . 5763632

[39] Maroti Z, Katona M, Orvos H, Nemeth I, Farkas I, Turi S. Heme oxygenase-1 expression in premature and mature neonates during the first week of life. Eur J Pediatr. 2007;166(10):1033–8. 10.1007/s00431-006-0375-x. . 17203280

[40] Colombatti R, Sainati L, Trevisanuto D. Anemia and transfusion in the neonate. Semin Fetal Neonatal Med. 2016;21(1):2–9. 10.1016/j.siny.2015.12.001 . 26732078

[41] Douglas CG, Haldane JS, Haldane JB. The laws of combination of haemoglobin with carbon monoxide and oxygen. J Physiol. 1912;44(4):275–304. 16993128 10.1113/jphysiol.1912.sp001517 PMC1512793

[42] Westphal M, Weber TP, Meyer J, von Kegler S, Van Aken H, Booke M. Affinity of carbon monoxide to hemoglobin increases at low oxygen fractions. Biochem Biophys Res Commun. 2002;295(4):975–7. . 12127991 10.1016/s0006-291x(02)00781-7

[43] Stark MJ, Clifton VL, Wright IM. Carbon monoxide is a significant mediator of cardiovascular status following preterm birth. Pediatrics. 2009;124(1):277–84. 10.1542/peds.2008-0877 . 19564310

[44] Henry E, Christensen RD. Reference Intervals in Neonatal Hematology. Clin Perinatol. 2015;42(3):483–97. 10.1016/j.clp.2015.04.005 . 26250912

[45] Sharma VS, Schmidt MR, Ranney HM. Dissociation of CO from carboxyhemoglobin. J Biol Chem. 1976;251(14):4267–72. . 6474

[46] Christou H, Bailey N, Kluger MS, Mitsialis SA, Kourembanas S. Extracellular acidosis induces heme oxygenase-1 expression in vascular smooth muscle cells. Am J Physiol Heart Circ Physiol. 2005;288(6):H2647–52. 10.1152/ajpheart.00937.2004 . 15681695

[47] Schaer DJ, Buehler PW, Alayash AI, Belcher JD, Vercellotti GM. Hemolysis and free hemoglobin revisited: exploring hemoglobin and hemin scavengers as a novel class of therapeutic proteins. Blood. 2013;121(8):1276–84. Epub 2012/12/25. blood-2012-11-451229 [pii] 10.1182/blood-2012-11-451229 23264591 PMC3578950

[48] Schaer DJ, Vinchi F, Ingoglia G, Tolosano E, Buehler PW. Haptoglobin, hemopexin, and related defense pathways-basic science, clinical perspectives, and drug development. Front Physiol. 2014;5:415 10.3389/fphys.2014.00415 25389409 PMC4211382

[49] Meyer J, Prien T, Van Aken H, Bone HG, Waurick R, Theilmeier G, et al Arterio-venous carboxyhemoglobin difference suggests carbon monoxide production by human lungs. Biochem Biophys Res Commun. 1998;244(1):230–2. 10.1006/bbrc.1998.8244 . 9514911

[50] Yasuda H, Yamaya M, Nakayama K, Ebihara S, Sasaki T, Okinaga S, et al Increased arterial carboxyhemoglobin concentrations in chronic obstructive pulmonary disease. Am J Respir Crit Care Med. 2005;171(11):1246–51. 10.1164/rccm.200407-914OC . 15764730

[51] Widdop B. Analysis of carbon monoxide. Ann Clin Biochem. 2002;39(Pt 4):378–91. . 12117442 10.1258/000456302760042146

[52] Coburn RF. The measurement of endogenous carbon monoxide production. J Appl Physiol (1985). 2012;112(11):1949–55. 10.1152/japplphysiol.00174.2012 . 22442030

